# Four-layer ConvNet to facial emotion recognition with minimal epochs and the significance of data diversity

**DOI:** 10.1038/s41598-022-11173-0

**Published:** 2022-04-28

**Authors:** Tanoy Debnath, Md. Mahfuz Reza, Anichur Rahman, Amin Beheshti, Shahab S. Band, Hamid Alinejad-Rokny

**Affiliations:** 1 Department of Computer Science and Engineering, Mawlana Bhashani Science and Technology University, Tangail, Bangladesh; 2 Department of Computer Science and Engineering, National Institute of Textile Engineering and Research (NITER), Constituent Institute of Dhaka University, Savar, Dhaka 1350 Bangladesh; 3 Department of Computing, Macquarie University, Sydney, NSW 2109 Australia; 4 Future Technology Research Center, CollegeofFuture, National Yunlin University of Science and Technology, 123 University Road, Section 3, Douliou, Yunlin 64002 Taiwan; 5 BioMedical Machine Learning Lab (BML), The Graduate School of Biomedical Engineering, UNSW Sydney, Sydney, NSW 2052 Australia; 6 UNSW Data Science Hub, The University of New South Wales (UNSW Sydney), Sydney, NSW 2052 Australia; 7 Health Data Analytics Program, Department of Computing, AI-Enabled Processes (AIP) Research Centre, Macquarie University, Sydney, 2109 Australia

**Keywords:** Computer science, Information technology, Engineering

## Abstract

Emotion recognition is defined as identifying human emotion and is directly related to different fields such as human–computer interfaces, human emotional processing, irrational analysis, medical diagnostics, data-driven animation, human–robot communication, and many more. This paper proposes a new facial emotional recognition model using a convolutional neural network. Our proposed model, “ConvNet”, detects seven specific emotions from image data including anger, disgust, fear, happiness, neutrality, sadness, and surprise. The features extracted by the Local Binary Pattern (LBP), region based Oriented FAST and rotated BRIEF (ORB) and Convolutional Neural network (CNN) from facial expressions images were fused to develop the classification model through training by our proposed CNN model (ConvNet). Our method can converge quickly and achieves good performance which the authors can develop a real-time schema that can easily fit the model and sense emotions. Furthermore, this study focuses on the mental or emotional stuff of a man or woman using the behavioral aspects. To complete the training of the CNN network model, we use the FER2013 databases at first, and then apply the generalization techniques to the JAFFE and CK+ datasets respectively in the testing stage to evaluate the performance of the model. In the generalization approach on the JAFFE dataset, we get a 92.05% accuracy, while on the CK+ dataset, we acquire a 98.13% accuracy which achieve the best performance among existing methods. We also test the system’s success by identifying facial expressions in real-time. ConvNet consists of four layers of convolution together with two fully connected layers. The experimental results show that the ConvNet is able to achieve 96% training accuracy which is much better than current existing models. However, when compared to other validation methods, the suggested technique was more accurate. ConvNet also achieved validation accuracy of 91.01% for the FER2013 dataset. We also made all the materials publicly accessible for the research community at: https://github.com/Tanoy004/Emotion-recognition-through-CNN.

## Introduction

**Figure 1:**
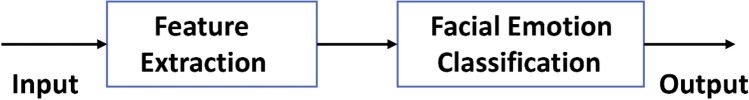
A simple structural view of facial expression recognition system.

The face is also known as the mental core. As an assortment of facial gestures, the face can give several minimal signals. These exquisite signals can make human–machine interaction more secure and harmonious when interpreted by computers. A good source of knowledge for ordering an individual’s true emotions^1^ was argued for facial expressions. Recognition of facial expression (FER) is one of the most critical non-verbal processes by which human–machine interface (HMI) systems can understand^2^ human intimate emotions and intentions. This scheme is a classification task. The classifier takes as input a set of characteristics that are derived from the input image, which is simply shown in Fig. 1.

Gabor wavelet transform^3^, Haar wavelet transform^4^, Local Binary Pattern (LBP), and Active Presence Models (AAM)^5^ are the feature extraction methods based on static images. Whereas dynamic-based^6–8^ approaches assume the temporal association in the sequence of input facial expression within clinging frames. Support Vector Machine (SVM), Hidden Markov Model, AdaBoost, and Artificial Neural Networks (ANN)^9^ are widely used schemes for facial expression recognition. A major advancement in the field of deep learning and the implementation of CNN has been quite promising^10–12^. However, a massive issue with the use of deep learning is that a large amount of data is required to learn successful models.

While some improvement in the identification of facial expression has been made by the CNN algorithm, some detachments are still present, including too long training times and low recognizing rates in the complex environment. In existing databases, two challenges have been observed in deep learning achievements in FER methods: (1) a low number of images, and (2) images taken from heavily structured conditions. These concerns inspired the creation of FER techniques focused on the set of Web images^13,14^. The present work focuses mainly on the creation of a multimodal, intelligent HMI system that operates in a real-time context.

This research aims to determine the emotion of a facial emotional input image. In this paper the authors do a more reliable and detailed study on deep learning both for static and dynamic FER tasks until 2020. This study is concerned with the creation of an automated facial expression recognition (AFER) system in the domain of facial expression using Convolutional Neural Networks (CNNs) and improving the accuracy of this mission. Orthodox machine learning algorithms used for handcrafted features typically have equivalents that do not have the durability to reliably interpret a task^15^. This is a fair starting point for us to examine the use of fused LBP-ORB and CNN features, since with CNN-based models^16^, we have obtained the best solutions to recent FER-relevant tasks.

Facial recognition requires several phases: detection of face images, preprocessing of face images, retrieval of facial features, alignment of face images, and identification of face images. There are primarily two types of extraction of features: one is geometric attribute extraction, and the other is a procedure which focused on total statistical characteristics. To describe the location of facial organs as the features of the classification^17^, the geometrical feature-based approach is widely used.

This paper aims at creating a method for the use of CNN to build a FER scheme. The presented model can be used in real-time using a webcam to categorize human faces. The contributions to this paper are as follows:

- The authors suggest a CNN method for recognizing seven facial expressions and real-time detection of facial expressions using the fusion of Convolutional Neural Network (CNN), Local Binary Pattern (LBP) features and Oriented FAST and rotated BRIEF (ORB).
- The authors propose a four-layer ‘ConvNet’ model for this system with the best uses of CNN parameters.
- This research reveals that combining images from different databases helps to increase generalization and to improve the accuracy of teaching.
- It can contain enhanced testing techniques, such as preparation, testing, and validating processes, and provides findings that reflect greater consistency by longer training sets instead of training and testing sets.
- The performance of the “ConvNet” architecture is evaluated on both large and small datasets. Our system appears to be capable of attaining excellent performance in both situations, according to the results.
- This work achieves a training accuracy of over 95% in a minimal number of epoch, showing that the model is well adjusted to the method. The classification accuracy obtained by the generalization techniques with three datasets are 91.05%, 92.05% and 98.13%, respectively.

This work aims to create a model that can classify seven distinct emotions: happy, sad, surprise, angry, disgust, neutral, and fear, and to achieve better accuracy than the baseline^18^. Besides this, the main goal of this research is to examine and understand the advantages of using deep convolutional neural network models over other deep learning models.

## Background knowledge and literature reviews

### Background study

#### Analyze of facial expression

**Figure 2:**
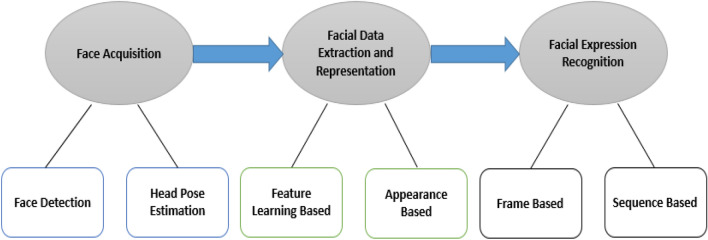
The basic framework of applications in many areas for automatic facial expression analysis.

Automatic facial expression analysis (AFEA) can be used in many areas, including relational and semantic communication, clinical psychology, psychiatry, neurology, pain assessment, lie detection, intelligent settings, and multimodal human–computer interface (HCI). Face collection, facial data extraction and representation, and recognition of facial expression are three steps of the standard approach to AFEA composition, as depicted in Fig. 2. There are mainly two types of techniques for facial feature extraction: geometric or predictive feature-based methods and methods based on appearances. The authors used the combination of statistical appearance-based and geometric feature-based approaches in this article.

#### Facial emotion recognition (FER)

**Figure 3:**
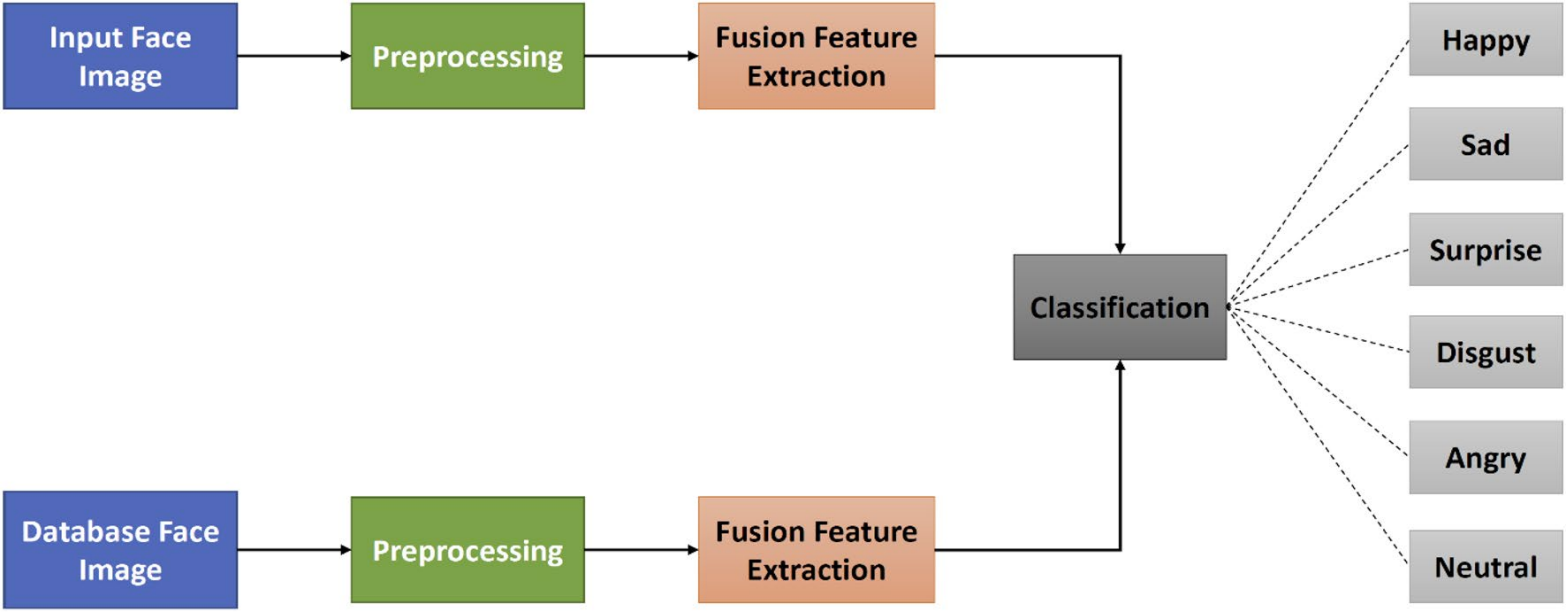
Summary flowchart for the three phases of the Facial Expression Recognition (FER) method.

Face detection is a key role in FER. There are different strategies to face recognition, including the expression-based approach, the framework approach, the feature-based approach, and the neighborhood graph approach^19^. The three-stage flow map of the facial expression recognition process seen in the Fig. 3.

#### Structure of CNN

**Figure 4:**
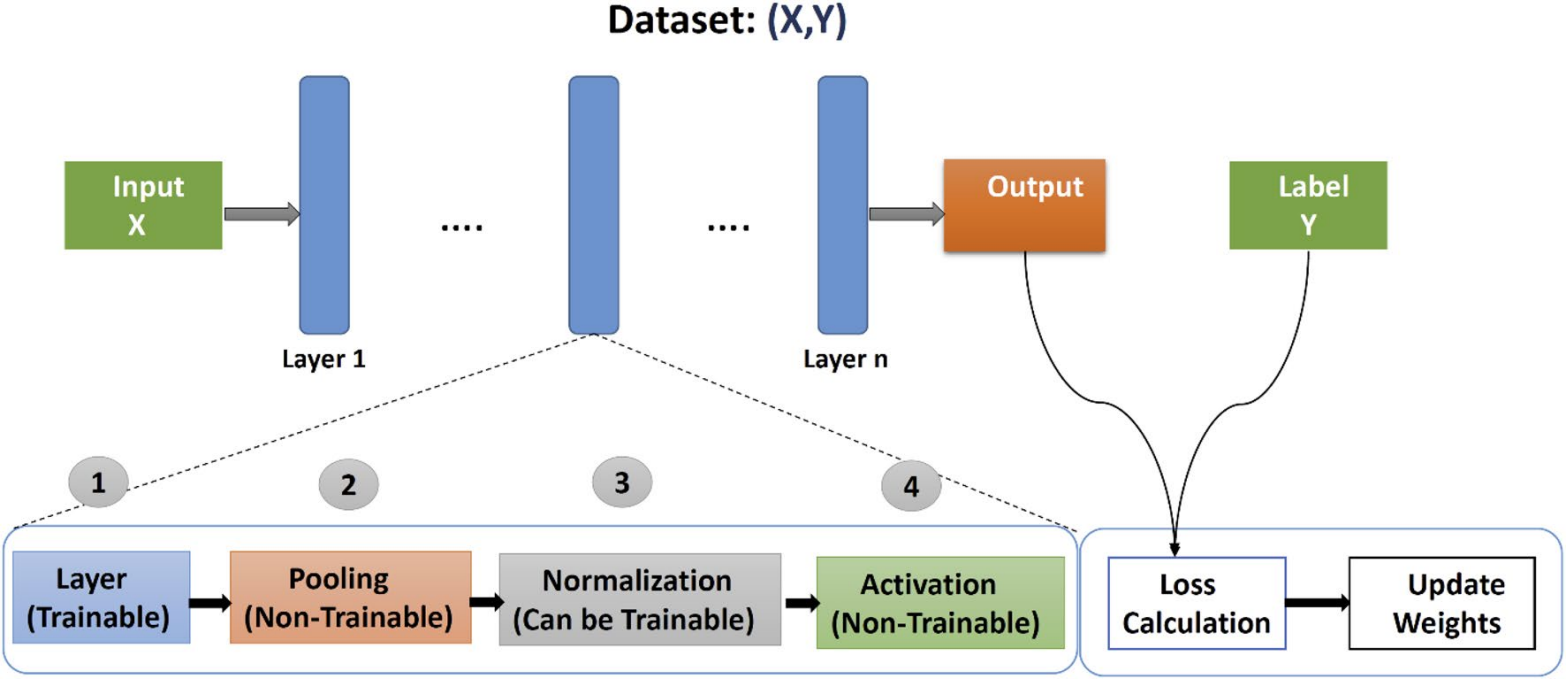
The template for a basic CNN (a simple CNN template with several building blocks).

At first, we see a simple CNN template with several building blocks that we can easily understand and correlate to the proposed CNN model. Three types of layers make up a basic CNN as illustrated in Fig. 4, input, hidden, and output. The data enters the CNN via the input layer and then travels through many hidden levels before reaching the output layer. The network's prediction is reflected via the output layer. In terms of loss or error, the network's output is compared to the actual labels.

The hidden layers in the network act as a basic building element for data transformation. The four sub-functions: layer function, Pooling, Normalization and Activation can be dissected from each layer. The Convolutional neural network architecture consists of the following layers^20^. We also discuss the CNN model parameters in the perspective of our study.

- Convolution layer

In the case of a Convolutional Neural Network, the primary goal of convolution is to extract features from the input picture. By learning image features (i.e., filtering with tiny squares of input data), convolution preserves the spatial relationship between pixels. The terms 'filter', 'kernel', and 'feature detector' are used by CNN. The 'Convolved Feature' or 'Activation Map' or 'Feature Map' is the matrix created by sliding the filter across the picture and computing the dot product. Filter size and stride (the number of pixels after which the filter should be changed) are selected in this layer. (W–F + 2P)/S + 1 = O is the output of the convolutional layer, where F, P, S means spatial extent, stride and padding respectively. Neurons do not "fit" smoothly and uniformly across the input if stride is not chosen properly. As a result, zero padding is utilized throughout the image in order to fit neurons properly.

- ReLu layer

Every negative value from the filtered picture is eliminated and replaced with zero in this layer. This is done to prevent the values from adding up to zero. The Rectified Linear Unit transform function only activates a node if the input exceeds a specific threshold; else, the output is zero. The value of the activation function of ReLu is that its gradient is always equal to 1 (shown in Eq. 1), meaning that during back-propagation, most of the error is transferred back^21,22^.

1 $$ f\left( x \right) = max\left( {0,x} \right) $$

- Pooling layer

A pooling layer's purpose is to minimize the spatial dimensionality of the corrected feature map, resulting in a more compact feature extraction. A pooled featured map is the result of this pooling layer. The two most common pooling strategies are maximum pooling and mean pooling. In the Max Pooling techniques, the maximum parameter is taken out at each step and the rest is lowered. Along with, the network is simply down-sampled by this. To calculate pooling layer, the formula is (I + 2P − F)/S + 1 = O, where I, S, P, F means input matrix, stride, padding and filter respectively.

- Fully connected layer

Fully Connected Layer remaps a pooled feature map from a two-dimensional structure to a one-dimensional vector, or feature vector. The result is a pooled feature map that has been "flattened". For classification, this feature vector acts as a standard Fully connected layer.

- Softmax

Softmax is implemented through a neural network layer just before the output layer. As the output layer, the Softmax layer must have the same number of nodes.

- Batch normalization

Batch normalizer speeds up the training process and adds a transition which keeps the mean activation near 0 and the standard activation deviation close to 1.

## Literature reviews

Facial communication studies have been carried out for years. But for any experiment, there was still room for progress. That is why this topic is convenient. The key objective of the researchers is to enhance the precision of a basic data collection FER2013 in Ref.^23^. The authors have used CNN as the methodology for their proposed model to define seven critical emotions. While overall accuracy has been obtained at 91%, the identification rate is only 45% and 41% respectively in classifying disgust and fear.

The authors of this paper^24^ propose a method that combines orientated FAST and rotated BRIEF (ORB) characteristics with facial expression-derived Local Binary Patterns (LBP) features. Finally, a Support Vector Machine is used to classify the combined characteristics (SVM). The suggested technique is tested on various difficult databases, including the Cohn–Kanade database (CK+), the Japanese Female Facial Expressions database (JAFFE), and the MMI database, with accuracy rates of 93.2%, 88.5%, and 79.8% respectively.

In Ref.^25^, the writers have identified facial expressions based on CNN. In comparison to other approaches, the proposed FER approach is a major challenge in machine learning, focused on mixed instances taken from multiple datasets. Experimental results reveal that the six universal expressions can be specifically defined by the FER process. In the recent past, a multimodal speech emotion recognition and classification techniques have been proposed by Christy et al.^26^. For classification and prediction, algorithms such as linear regression, decision tree, random forest, support vector machine (SVM) and convolutional neural networks (CNN) are used in this article. The authors tested their model with the RAVDEES dataset and, compared to decision tree, random forest and SVM, CNN showed 78.20% accuracy in identifying emotions.

In the discussion of the prediction system or algorithm, the authors^27^ used a large milking dataset to test multiple machine learning-based prediction algorithms to find the best predictive models of sub-clinical mastitis. The Z transformation approach was used to offer a platform for the models developed to be applied to additional farms. The non-transformed milking dataset and a Z-standardized dataset were both subjected to several prediction methods. Gradient-Boosted Tree (GBT) and Deep Learning (DL) outperform other models in these systems. In terms of predicting subclinical bovine mastitis, GBT was the most accurate model (with an accuracy of 84.9 percent). These results show how these models may be used to predict subclinical mastitis in a variety of bovine herds, regardless of size or sampling technique.

Though there are some limitations to this study, such as the models not being converted to a web application for easy access and use by biologists and clinicians, it does show that the two prediction models (GBT and DL) can accurately forecast sub-clinical mastitis based on multiple milking variables, confirming the power of machine-based prediction in solving one of the oldest problems in the dairy cattle industry. In a residual block based CNN method, the first part of this work^28^ introduces a unique idea of electrode-frequency distribution maps (EFDMs) based on a short-time Fourier transform (STFT). For automated feature extraction and emotion detection with EFDMs, a residual block based deep convolutional neural network (CNN) is suggested. Based on a short length of EEG data on SEED, the suggested technique earned an average classification score of 90.59%, which is 4.51% higher than the baseline method. Then, using deep model transfer learning and a few data, the pre-trained model was applied to DEAP, yielding an average accuracy of 82.84%.

Wang et al.^29^ proposed a novel concept of EFDMs with STFT based on multiple channel EEG signals. The pre-trained model was then introduced to DEAP with a few samples by profound model transmission, resulting in 82.84% accuracy on average. Jung and associates^7^ investigated FER with a profound learning approach, which integrates two deep networks that derive faces appearance (using convolutional layers) and geometric features from face landmarks (using completely linked layers), with a 97.3% accuracy of CK+ findings. The authors suggested a computer vision FER method in Ref.^30^. In the process, the gray-scale face picture was consolidated into a 3-channel input with the corresponding basic LBP and an average LBP feature map. This thesis won the Emotiw 2017 award with the best submission reaching 60.34% accuracy. Francesca Nonis et al.^31^ suggested 3D approaches and problems in FER Algorithms. This research would address the problem of facial identity through the interpretation of human feelings, focusing on 3D approach, grouping and arranging all the works and different techniques. The average accuracy of recognition of expressions varies from 60 to 90%. Certain expressions, such as anger and fear, have usually the lowest recognition levels.

Smitha Rao et al.^32^ recently proposed a CNN-LSTM based Neural Network for six fundamental emotions, Angry, Happy, Sad, Fear, Disgust, and Neutral, which was trained on the CREMA-D dataset and evaluated on the RAVDEES dataset. The study focuses on the usage of LSTM networks, which are capable of employing a sequence of data to help in the overall prediction of emotions in a movie. On the CREMA-D dataset, they attained an accuracy of 78.52%, while on the RAVDEES dataset, they achieved an accuracy of 63.35%.

A facial expression recognition system has been introduced by N. Veeranjaneyulu^33^ in which facial characteristics by use of deep neural features much better than handcrafted ones are. The extraction function is conducted using the VGG16 algorithm and deep CNN models are classed. The suggested accuracy of the system is demonstrated by the CK+ dataset. In Ref.^34^, the authors have introduced a 3-dimensional neural video emotion detection network. The findings are contrasted with cross-validation approaches. The crafted 3D-CNN generates 97.56% with the cross-validation of Leave-on-Sujet-Out, and 100% with 10 times CK+ and 84.17% with 10 times Cross-validation on Oulu-CASIA. Deep learning methods for facial recognition are presented in this paper^35^ for effective identification and detection. The face characteristics, on the other hand, are collected in real time and analyzed with haar cascade detection. Face detection, identification, and emotion classification are the three main aims of the proposed study. Finally, using the KDEF dataset, the accuracy of automated face identification and recognition is assessed at 63.88%.

The analysis of facial expression recognition has some drawbacks, according to the authors. Such as the model’s use and lack of friendliness, inability to catch feelings or actions in complex contexts, participant shortness with a need for more accuracy, a deficit in detecting effectiveness about EEG signals, and so on. Although there has been more research on combining impact identification and usability testing, their functional applicability and interference with user experience testing still need to be analyzed further. In Ref.^36^, without needing any pre-processing or feature extraction tasks, the authors demonstrate the classification of FER based on static images, using CNNs. In a seven-class classification assignment, the authors obtained a test accuracy of 61.7% on FER2013 compared to 75.2% in the state-of-the-art classification.

## Proposed architecture and methods

Convolutional Neural Networks are a form of deep neural network that is used for computer vision and visual image processing. However, conventional algorithms face certain serious issues or questions, such as luminous variance and location variance, etc. The approach to addressing the problems of conventional methods is to apply the CNN algorithm to the classification of emotions. In contrast, our model is sequentially structured. We recognize that Sequence Modeling has the ability to model, analyze, make predictions or produce some form of sequential data. In comparison to the traditional form, the algorithm’s major distinctions are:

1. Feature extraction with LBP
  With the use of Local Binary Pattern (LBP), image feature map can be extracted, as the feature extractors are generated during the training and testing process.
2. Differences of mathematical model
  Typically, the linear classifier is classified by linear transformation. This is commonly referred to as the traditional form. In contrast, to discern variations in the classification process, CNN and other deep learning algorithms usually incorporate linear conversion with nonlinear features such as sigmoid and rectified linear unit functions (ReLU). We also take the advantages of strides and padding parameters in our model architecture.
3. The deeper structure

The traditional approach usually conducts only one layer of an operation via the linear classifier: SVM has just one weight set, for instance (shown in Eq. 2). However, in the course of classification, CNN and other deep learning algorithms perform several layers of operation. As a two-dimensional array, CNN adopts input.

2 $$ S = W \times x_{i} + b $$

where the classification score is S, W is the matrix of weights, and b is bias.

## Local binary pattern

Local Binary Pattern (LBP) is a term used to describe the texture properties of pictures on a local level. The major benefit of the Local binary pattern is its rotation and gray invariance. LBP is a basic technique for detecting characteristics in an image that is resistant to lighting fluctuations. It is a commonly utilized approach for feature extraction in various object identification and facial expression detection algorithms because to its simplicity and durability.

The Local Binary Pattern is an effective texture classifier. The binary operator labels the pixels in the image by comparing the center pixel value with the 3 × 3 neighborhood of each pixel value to create an 8-bit binary number, which is then transformed to a decimal value. The binary-valued picture patch that results is used to create a local image descriptor^37^. The following equation is used to calculate LBP code for a given pixel at (_**Xc, Yc**_):

3 $$\mathrm{LBP P},\mathrm{R }(\mathbf{X}\mathbf{c},\mathbf{Y}\mathbf{c}) = \sum_{{\varvec{n}}=0}^{7}{2}^{\begin{array}{c} \\ n\end{array}}{\varvec{S}}\boldsymbol{ }(\mathbf{i}\mathbf{n}-\mathbf{i}\mathbf{c}), {\varvec{S}}\left({\varvec{x}}\right)=\left\{\begin{array}{c}1, x\ge 0\\ 0, x<0\end{array}\right.$$

where, i_c_ = gray value of center pixel**,** i_n_ = gray value of neighboring pixel of i_c_**,** P = 8 maximum of 8 neighbors of center pixel.

$$ {\text{R }} = { 1}\,{\text{for}}\,{\text{selected}}\,{\text{box}}\,{\text{of}}\,{3} \times {3}{\text{.}} $$

**Figure 5:**
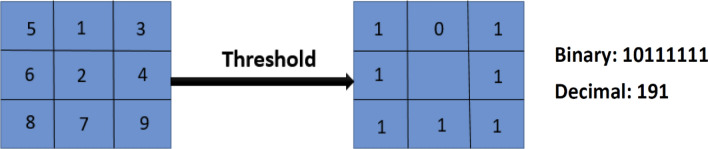
The basic LBP operator which labels the pixels in the image.

**Figure 6:**
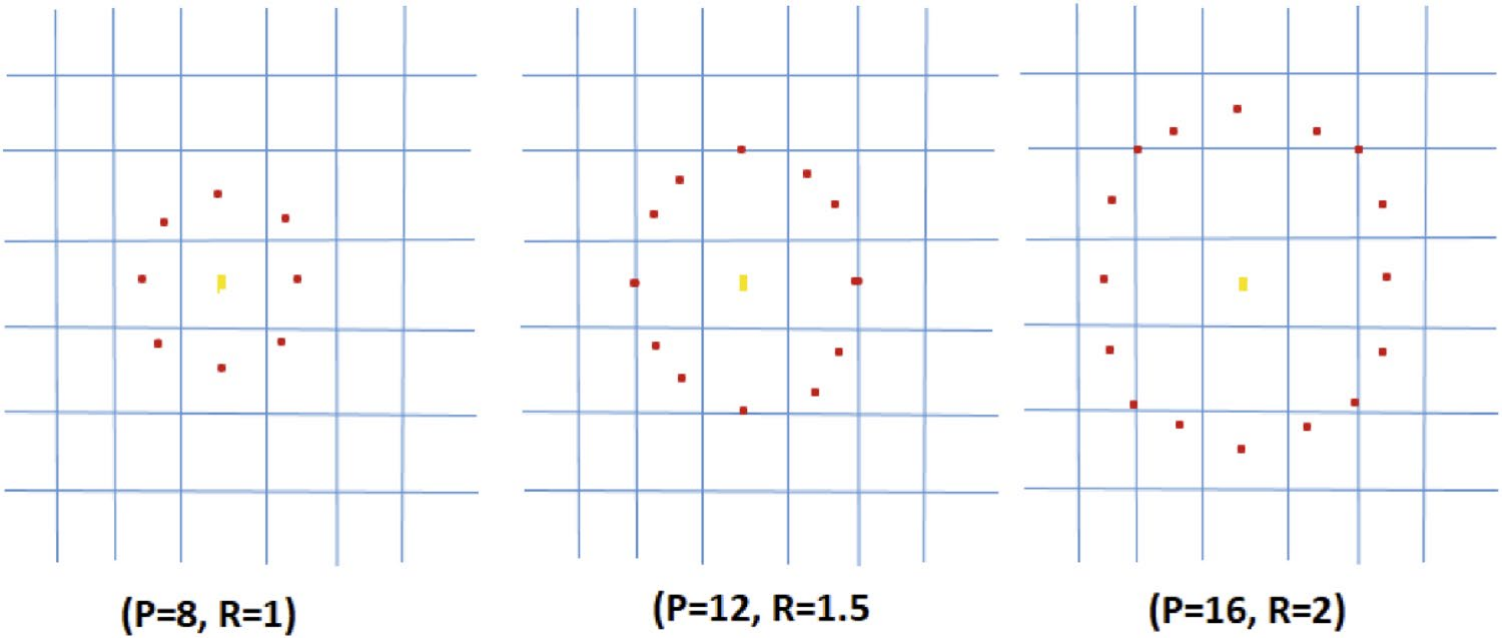
Three examples of the extended LBP which makes it ideal for real-time applications.

As a result, a pixel may have a total of 2^8^ = 256 distinct values assigned to it. For the center pixel's 8 neighbors, and are the gray values at and, respectively, and is 1 if and 0 otherwise. An original LBP operator that is shown in Fig. 5:

The LBP code is obtained by tracing the bins in a clockwise circle. These feature vectors serve as the classification process's input. Even if the lighting circumstances change, the relative pixel difference between the central pixel and the adjacent pixels remains constant in LBP, which is the major advantage of efficient LBP. This feature of the LBP makes it ideal for real-time applications. In Fig. 6, the notation (P, R) represents a neighborhood of P evenly spaced sampling points on a circle of radius R that constitute a circularly symmetric neighbor set. The second specified uniform patterns: an LBP is considered ‘uniform' if it has no more than one 0–1 and one 1–0 transition when examined as a circular bit string. For example, the numbers 00000000, 001110000, and 11100001 are all uniform patterns.

After using the LBP operator to label an image, a histogram of the labeled image fl (x, y) may be defined as follows:

4 $$ {\text{Hi}} = \sum {\text{x}},{\text{ yI }}\left( {{\text{fl}} \left( {{\text{x}},{\text{ y}}} \right) \, = {\text{i}}} \right), {\text{i}} = 0,{1}, \ldots ,{\text{ n}} - {1} $$

where n is the number of distinct labels generated by the LBP operator, and:

5 $${\varvec{I}}\left(A\right)=\left\{\begin{array}{c}1, A is true\\ 0, A is false\end{array}\right.$$

### Region-based ORB

Traditional ORB extracts a large number of feature points to improve accuracy, but the excessively dense feature points are difficult for feature description. To tackle this challenge, we used region division in the classic ORB in a unique way. The number of feature points to be extracted for each region is calculated using the total number of feature points to be extracted and the number of regions to be divided in this updated ORB algorithm. The following are the measures to take:

- Divide the pictures evenly into X × Y areas of equal size. The division's row and column are denoted by X and Y, respectively. The feature points are scattered at random in the separated regions, which are labeled {h1, h2,…, h_X×Y_}.
- Set a threshold T.
  6 $$\mathrm{T}=\frac{{\varvec{n}}}{{\varvec{X}}{\varvec{Y}}}$$
  where, the number of feature points is n
- In each region, feature points are recognized; if the number of features is greater than T, T is chosen as the feature number. If the number of features is less than T, lower the threshold and repeat the process.
- When the number of feature points is more than n, the best feature points are chosen using the nonmaximal suppression approach.
- The number of feature points must meet the conditions before all regions are covered.

### CNN model overview

**Figure 7:**
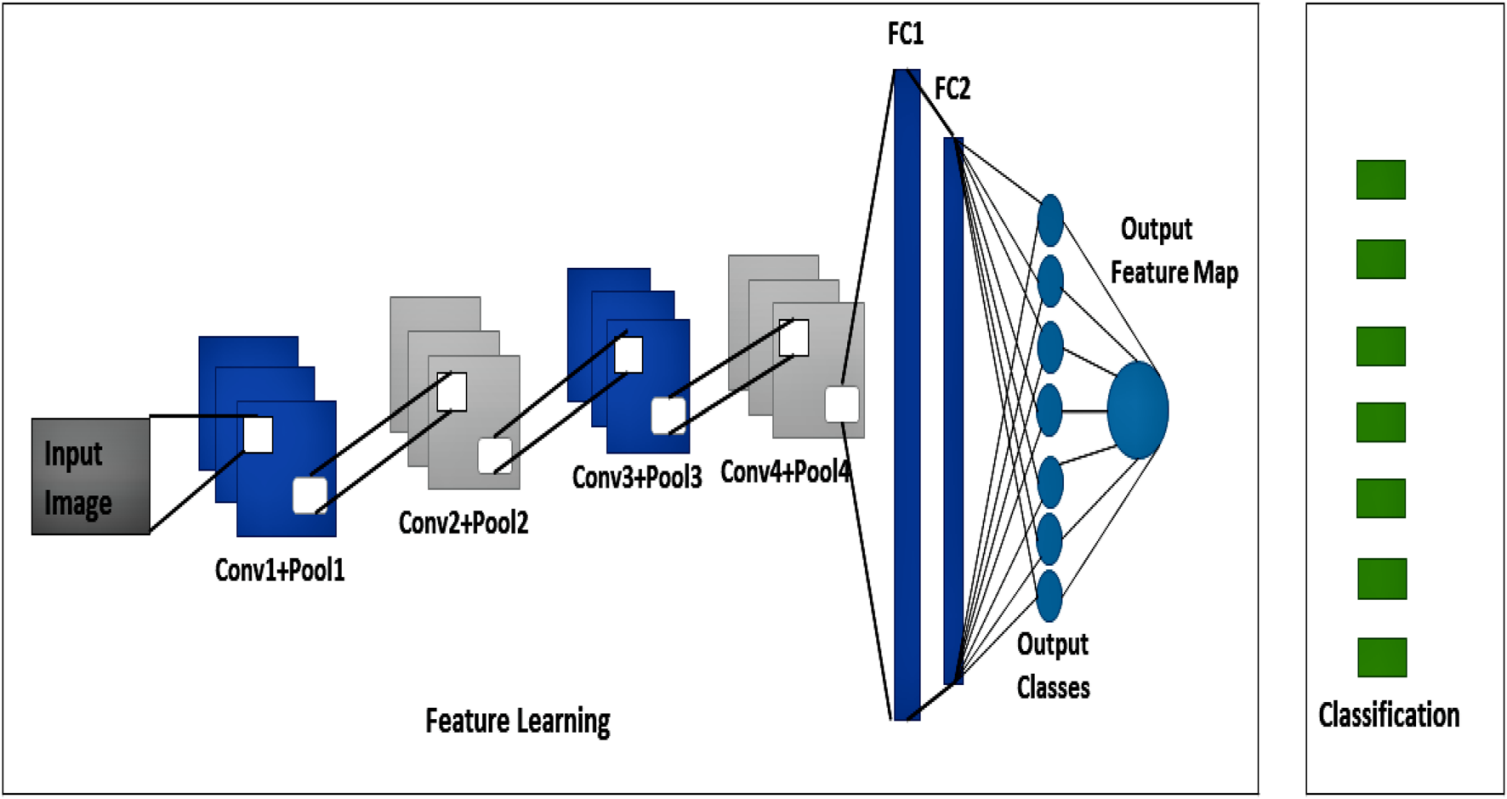
The graphical representation of the proposed CNN model for facial expression recognition.

The proposed model consists of four layers of convolution together with two layers that are fully connected which we can see in the Fig. 7. The vectors that we acquire feature vectors after convolution from each filter. The LBP feature map will be fused with this feature vector to create a combined convoluted feature map. In addition, the convolution layer has weights that need to be taught, whereas the pooling layers use a fixed function to convert the activation. The Rectified Linear Unit (ReLU) is used to provide non-linearity to the entire network while having no effect on the convolutional layer's receptive fields. The efficiency of the convolution layer goes through loops. Furthermore, model output is calculated on a training dataset with the loss feature and learning parameters (kernels and weights) are adjusted by back-propagation with the loss. This work requires to incorporate or delete certain layers during training cycles, such as Max Pooling or Convolution layer, to build something unique and useful under specific kernels and weights for the output of a model. The output is pooled, which is basically a non-linear down-sampling process in the spatial dimensions.

Pooling lowers, the representation's spatial size, which helps to reduce parameters and calculations, and therefore control over-fitting. Finally, two completely connected layers are utilized to remap the pooled feature map from a two-dimensional structure to a one-dimensional vector, or feature vector. The result is a pooled feature map that has been "flattened." For classification, this feature vector serves as a standard Fully connected layer.

#### Fine tuning for proposed CNN

Not only does a fine-tuning methodology replace the pre-trained model’s fully connected layers with a new set of fully connected layers to train up on a given dataset, but it also fine-tunes all or part of the kernels in the pre-trained convolutional layer base by way of backpropagation. The hyper-parameters that control the output size of the convolutional layer are padding, stride, batch size, filter, sliding window and learning rate parameter. Padding is required to add zeros to the border of the input. The allocation of width and height parameters is controlled by stride. Small stride sizes lead to quite heavily over-lapping receptive fields and large output. The receptive fields overlap less with larger strides, resulting in output with smaller dimensions. It is possible to fine-tune all the layers in the convolutional base or set some earlier layers while fine-tuning much of the deeper layers. In this work, the proposed model consists of four layers of convolution together with two layers that are completely connected. This task would only need to train the high-level detailed feature block the essence and the completely connected layers that regard as a classifier. In contrast, since we have just 7 emotions, the authors reset the Softmax ranking to 7 grades from 1000 ranks.

#### Pipeline for proposed CNN

**Figure 8:**
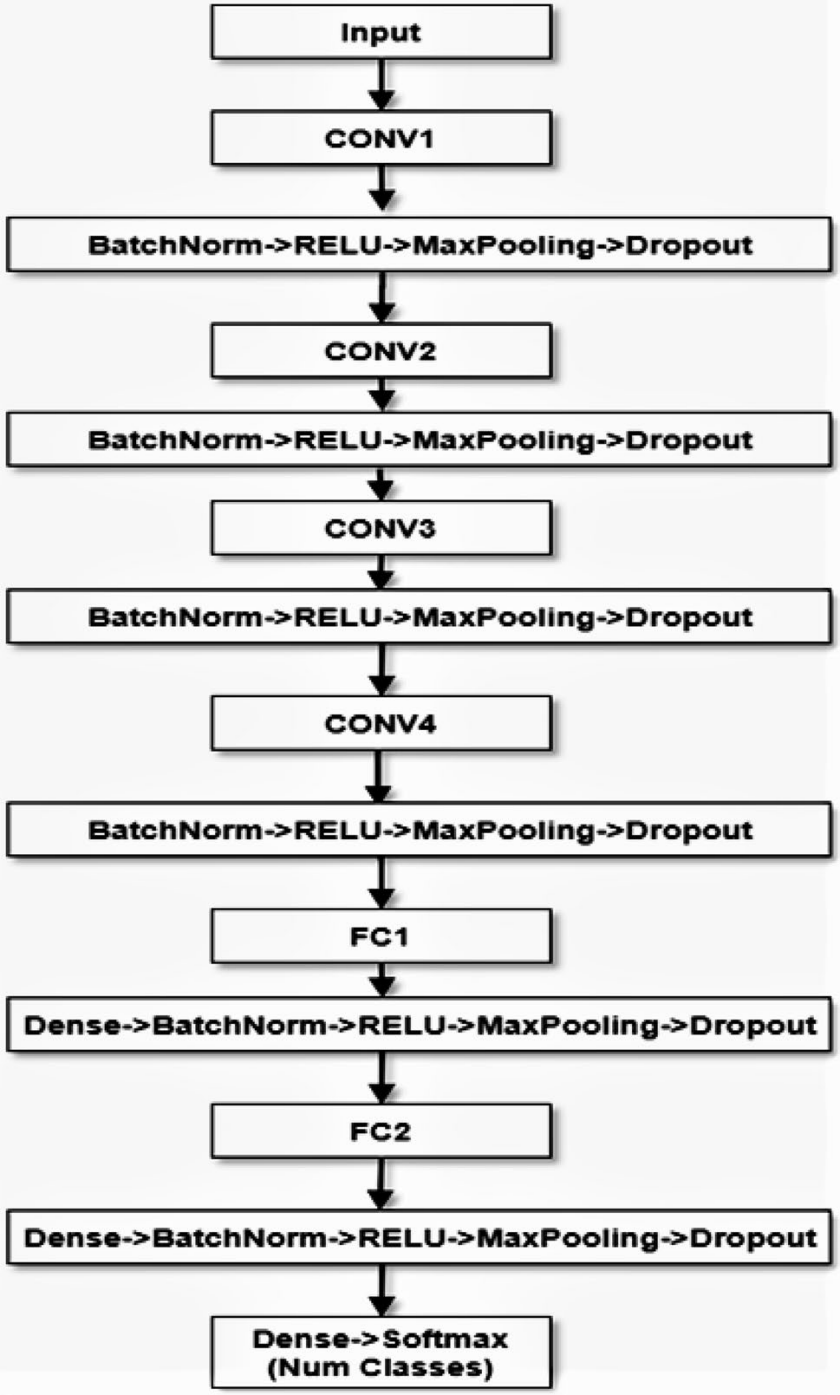
The structural pipeline of the proposed CNN model for facial expression recognition.

The network with a layer for processing the input. Here are four convolution and additional pooling layers and two fully connected layers that is completely associated at the end. A ReLU layer, batch normalization, and a dropout layer is used for any convolution and a fully connected layer of all the four network structures. The additional dense layer is used at the end of the four convolution layers which are associated with the two fully connected layers. Besides, the overall pipeline for the proposed CNN model is architectured on the following Fig. 8.

## Proposed methodology

### Datasets

For our purposes, we can use the Extended Cohn-Kanade (CK +), JAFFE, and FER2013 Dataset. The FER2013 dataset, on the other hand, has a resolution of just 48 × 48. As a result, it may be used to compare the performance of both methods in low-light situations. It also includes a dedicated public and private dataset for validation and testing. JAFFE and FER2013 datasets have Grayscale image. On the other hand, both RGB and Grayscale images are available in CK+. For the sake of simplicity, all of them must be in grayscale.

### FER2013 database

**Figure 9:**
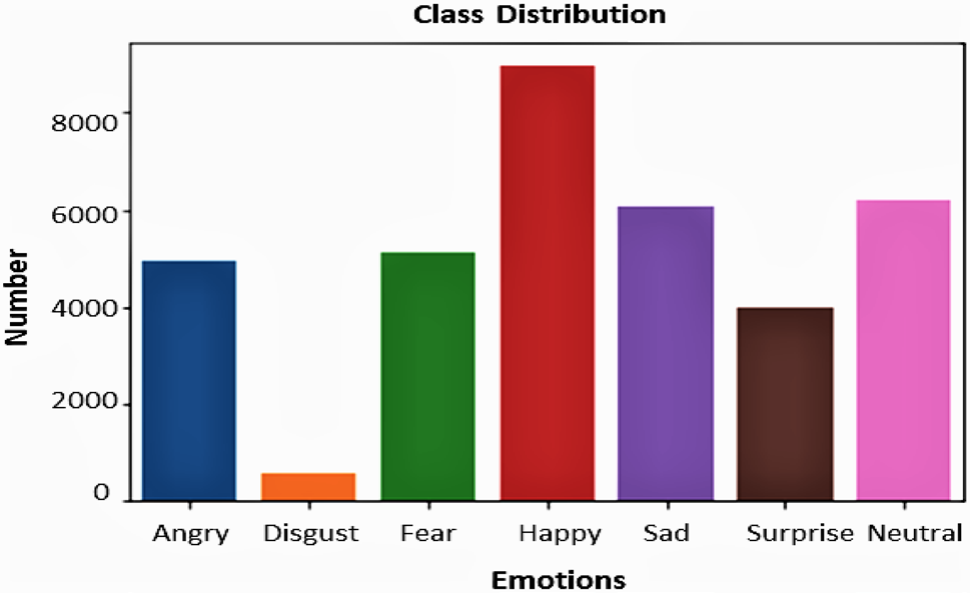
Seven facial expressions distribution of the FER2013 dataset.

The data collection used for the application was the FER2013 dataset from the Kaggle challenge on FER2013^38^. The database is used to incorporate the Facial Expression detection framework. The dataset consists of 35,887 pictures, split into 3589 experiments and 28,709 images of trains. The dataset includes another 3589 private test images for the final test. Figure 9 shows the expression distribution of the FER2013 dataset.

**Figure 10:**
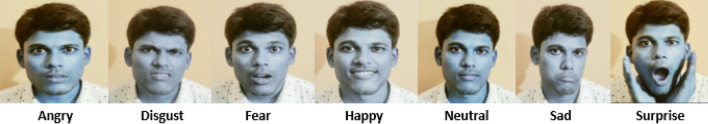
The examples of seven basic emotions for the validation purpose. . (Figure adapted from our own test images^39^)

We have created some real-time own test images for recognizing emotions. Figure 10 shows the examples of seven basic emotions from the own test image dataset^39^.

### Japanese female facial expressions (JAFFE) database

JAFFE's database has 213 pictures, each with 3 or 4 sample instances of 7 facial emotions (six fundamental facial emotions and one neutral emotion) from 10 distinct Japanese female participants with a resolution of 256 × 256^40^, which are gray. In our experiment, we utilized a total of 213 pictures to assess the suggested algorithm (anger: 30 images; disgust: 29 images; fear: 32 images; happiness: 31 images; neutral: 30 images; sad: 31 images; and surprise: 30 images).

### The extended Cohn–Kanade (CK+) database

The CK+ database comprises 593 sequences from 123 people aged 18 to 30. Anger (45), neutral (18), disgust (59), fear (25), happiness (69), sadness (28), and surprise (83) are used to classify 327 sequences^41^. We selected 309 sequences from our trials that were classified as one of the six fundamental facial expressions, eliminating contempt.

The five major points of the proposed methodology are discussed here:

### Recognition of facial expression

**Figure 11:**
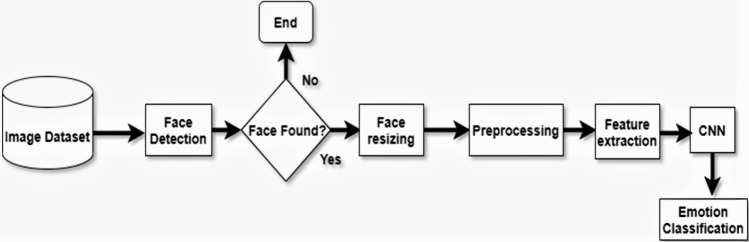
System flowchart of the proposed method (fusion feature extraction and fusion approaches with the proposed ConvNet Model) for emotion classification.

The FER mechanism has three steps. Firstly, the step of prepossessing is to prepare the dataset into a shape. The new form will run and produce effective results on a generalized algorithm. Secondly, the face is identified from the images collected in real-time in the feature extraction step. Finally, to group the picture into one of seven classes, the emotion classification stage consists of applying the CNN algorithm. Moreover, these main phases are represented using a flowchart. The system flowchart of emotion classification for the FER approach is seen in Fig. 11.

### Preprocessing

Preprocessing is a required step in computer vision for image processing. The picture entered in the dataset may include noise and some light, scale and color variations. Apart from this, any preprocessing operations in the Ref.^42^ picture have been performed in order to produce more reliable and quicker performance with the CNN approach. In the transformation of the image, the following preprocessing techniques are used:

- *Normalization* An image is normalized to eliminate differences and to produce an improved image of the face.
- *Gray scaling* Gray scaling is an image transformation method whose pixel value depends upon the strength of the image’s light. As colored images are hard to process by an algorithm, gray scaling is completed. Based on our knowledge with classifying facial expressions regardless of skin color, facial expression recognition using CNN must also be conducted independently of the color information of the input image. The input picture is merely converted to a 1-channel gray image in this article.
- *Redimensioning* The image is redimensioned to delete the unnecessary portion of the image. Undoubtedly, this decreases the required memory and increases the speed of calculation^43^.

### Face detection

**Figure 12:**
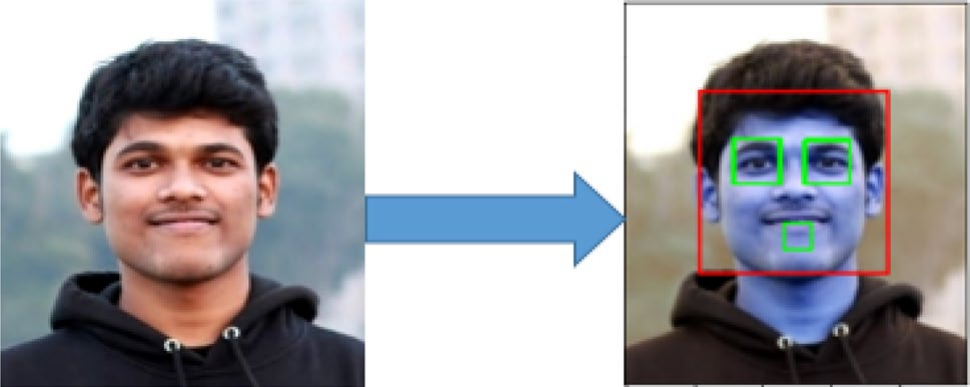
Different facial component selection of a happy image in a face detection process.

Face identification is the foundational step for every Facial Expression Recognition System. It is an efficient solution to object detection proposed in their article, “Rapid Object Detection using a Boosted Cascade of Simple Features”^44^ in 2001, by Paul Viola and Michael Jones. Classifiers that detect an object in a picture or video where many positive as well as negative images learn a cascade function. In addition, Haar cascades in images have proven to be a powerful means of object detection and provide high precision. Three dark regions on the forehead, such as the eyebrows, are detected by Haar features. Haar cascades delete the unwanted background data from the picture effectively and detect the face area from the picture. OpenCV introduced the face detection mechanism with Haar cascade classifiers. Facial Component detection of a happy image is seen in Fig. 12. This approach used rectangular characteristics^45^.

### Feature extraction

Because of its simplicity and durability, we utilize Local Binary Pattern as a feature extraction approach, which is extensively used for feature extraction in various object identification algorithms as well as facial expression recognition with efficient classification result. We see that “uniform” patterns make up the great majority of patterns for each image in our collection. The following steps are now used to implement feature extraction:

1. Split the image of the face into small segments.
2. For each region, calculate the LBP histogram. Each region's LBP histogram is obtained by scanning it with the LBP operator.
3. Combine the LBP feature histograms of each region into a single feature vector.

**Figure 13:**
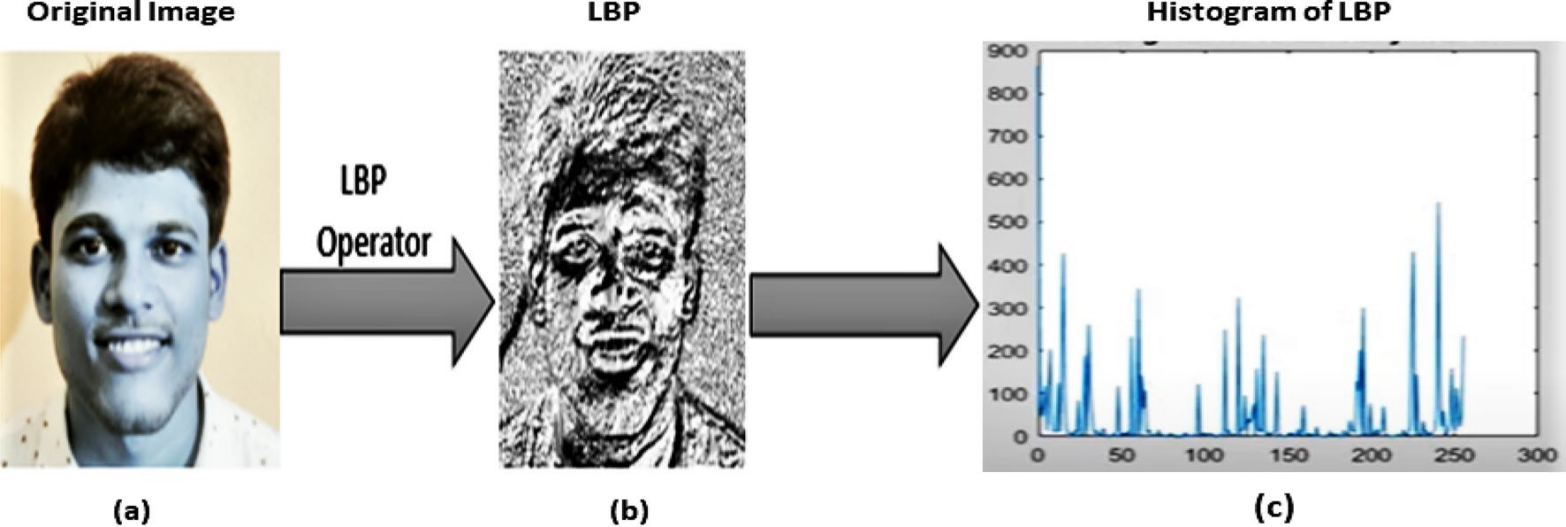
(**a**) The detected face from original image. (**b**) LBP of image. (**c**) Feature histogram of LBP image. . (Figure adapted from our own test images^39^).

**Figure 14:**
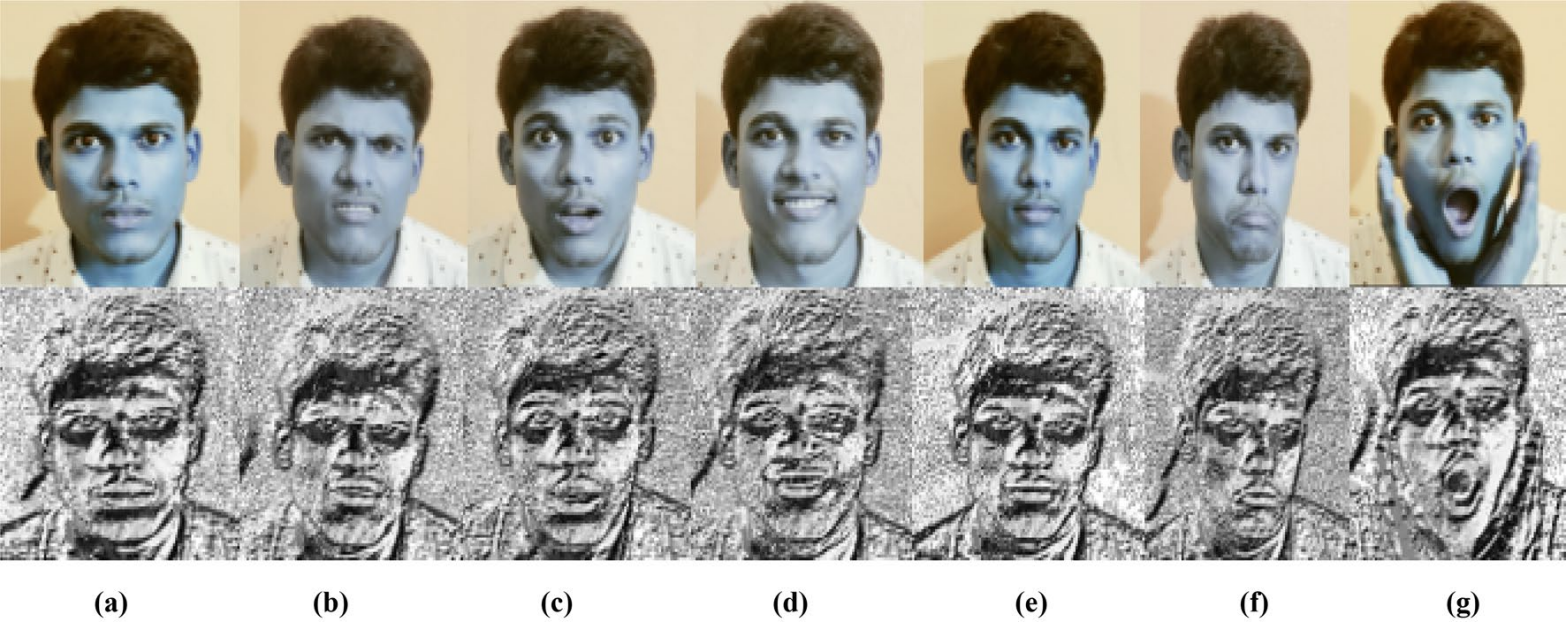
Original LBP features of facial expressions. (**a**) Happy (**b**) Angry (**c**) Fear (**d**) Sad (**e**) Disgust (**f**) Surprise (**g**) Neutral. . (Figure adapted from our own test images^39^).

The LBP histogram is a good indicator of changes in appearance as it combines information about shape, position and texture. The feature vector must be decreased for the classification to be faster based on linear programming approaches. Figures 13 and 14 indicates the LBP characteristics and how LBP operator can convert an original image to LBP image, and discovers an LBP image characteristic histogram.

### Scheme for feature fusion

Prior to feature fusion, feature normalization is used to improve the recognition rate. The LBP features are standardized to a range of 0–1 in this article, and the LBP and ORB features are normalized using the following formula:

7 $$\mathrm{L }=\frac{l}{\mathrm{max}(l)},$$

where, l denotes a feature's value. Since one type of feature cannot describe all visual characteristics, feature fusion is commonly used. The LBP and ORB descriptors are fused in this article using the Z-score approach.

$$\sigma =\sum_{j}^{J}{\left({f}_{i}-\mu \right)}^{2},$$

8 $$\upmu =\frac{\sum_{j}^{J}{f}_{j}}{J},$$

$${\widehat{f}}_{j}=K\frac{{(x}_{j-\mu })}{\sigma +C},$$

where, fj is an LBP or ORB feature, and ${\widehat{f}}_{j}$ is the fusion feature data. In the following trials, K is a factor multiplied by ${\widehat{f}}_{j}$, and K is 100.

#### Emotion classification

Here, the device classifies the picture into one of the seven universal expressions as entitled in the dataset—Happy, Sad, Anger, Surprise, Disgust, Fear, and Neutral. The training was carried out using CNN, which is a collection of neural networks. On the training range, the dataset was trained first. Before feeding it into CNN, the process of feature extraction was not performed on the results. The method followed was to experiment on the CNN with various architectures, to obtain better accuracy with the validation set. The step of classification of emotion consists of the following stages:

- Data splitting:
- The dataset was separated into three categories: training, public testing, and private testing. A training and public test set was used for the generation of a model and a private test set was used for the validation of the model.
- Model training and generation:
- The design of the neural network was addressed in-depth in the layout of CNN section earlier. Here we can see that the proposed model was set to the network and that after training on datasets, the model updates will be generated and applied to the previous structure with the. json file.
- Evaluation of model:
- The updates of the model produced during the training process were evaluated on the validation set consisting of 3589 images.
- Using the CNN model to classify test dataset as well as real-time images:
- The transfer learning theory can be used to recognize the emotion in images here in real-time. The model developed during the training phase consists of the corresponding weights and values that can be used to detect new facial expressions. Since the created model already contains weights, it can certainly be said that FER is faster for real-time pictures.

## Experiments and results analysis

### Accuracy and complexity

Since the proposed model has been trained on a composite dataset, training accuracy above 95% and validation accuracy above 91% has been reached, which would be 95% after performing several epochs. It can be mentioned earlier that just after 30 epochs, the CNN model has a training accuracy of 95%, whereas CNN has taken further epochs to reach greater accuracy. A slight comparison of the suggested approach with other related works is seen in the Table 1. From the table, it can be ensured that the CNN approach is much better than adjusting any other technique or approach to the recognition of human emotions, and our proposed model demonstrates better work.

**Table 1:** Accuracy Comparison with related network.

Algorithm	Accuracy (%)	Computational complexity
Alexnet^46^	55–88	${\text{O}}\hat{4}$
VGG^47^	65–68	${\text{O}}\hat{9}$
GoogleNet^48^	82–88	${\text{O}}\hat{5}$
Resnet	72–74	${\text{O}}\hat{1}6$
FER (our proposed)	75–96	${\text{O}}\hat{4}$

The performance of the system established for facial emotion recognition from images was measured using three metrics: accuracy, specificity, and sensitivity. The number of correctly classified data is divided by the total number of the data to calculate accuracy. True-positive (TP), true-negative (TN), false-positive (FP), and false-negative (FN) performance characteristics were used to compute the metrics as specified by Eqs. (9–11).

9 $$\mathrm{Accuracy }=\frac{TN+TN}{TP+FP+FN+TN},$$

10 $$\mathrm{Sensitivity }=\frac{TP}{TP+FN},$$

11 $$\mathrm{Specificity }=\frac{TN}{TN+FP}.$$

The classification accuracy is used to evaluate the system's overall effectiveness, which we focused on afterward employing generalization techniques and compared to state-of-the-art methodologies or measurement.

The authors also compared the computational complexity of the related network. The method of calculating the computational complexity of our proposed method in Table 1 is as:

We see that for each layer a matrix multiplication and an activation function are computed. We know that naive matrix multiplication has an asymptotic run-time of O(n^3^) and if g(x) is an activation function which is an element-wise function, we also know that it has a run-time of O(n). When analyzing matrix algorithms, it's common to assume that the matrices are quadratic; that is, they have the same number of rows as columns. By doing this, we find that:

12 $$ {\text{n}}\left( {{\text{mul}}} \right) \, = {\text{ n}}_{{{\text{layers}}}} \times {\text{n}}^{{3}} . $$

If we once again assume that there are the same number of neurons in each layer, and that the number of layers equal the number of neurons in each layer we find:

13 $$ {\text{n}}\left( {{\text{mul}}} \right) \, = {\text{ O }}\left( {{\text{n }} \times {\text{ n}}^{{3}} } \right) \, = {\text{ O }}({\text{n}}^{{4}} ). $$

The same can be done for the activations:

14 $$ {\text{n}}\left( {\text{g}} \right) = {\text{ n}}_{{{\text{layers}}}} \times {\text{n }} = {\text{ O}}\left( {{\text{n}}^{{2}} } \right). $$

So, for the proposed model, the total run-time becomes:

15 $$ {\text{O}}\left( {{\text{n}}^{{4}} + {\text{n}}^{{2}} } \right) \Leftrightarrow {\text{O}}\left( {{\text{n}}^{{4}} } \right)\quad \because \forall n \ge {1} {\mid }n^{{4}} + n^{{2}} \le {2}n^{{4}} . $$

In the other network, there are a number of layers present in the architecture. The number of minimum convolution layers are 8, 16, 22, 50 for AlexNet, VGG, GoogleNet, and ResNet respectively. So, in their perspective, the time complexity was calculated with the above approach.

The confusion matrix based on the FER2013 validation test dataset shown on below in Table 2. It can be seen that the accuracy for most expressions is well mannered. As the epoch increases in a consistent manner during each training cycle, the model will be well-suited and perform well during the real-time testing period. The bold numbers of each class indicate that the research data has been well categorized. In addition, the numbers on each side of the diagonal show the number of pictures that have been inappropriately listed. As these numbers are smaller than the numbers on the diagonal, it can be inferred that the algorithm performed properly and obtained state-of-the-art outcomes^43^.

**Table 2:** Confusion matrix of 7 class facial emotion recognition results obtained by ConvNet with LBP on the FER2013 validation test dataset.

	Angry (%)	Disgust (%)	Fear (%)	Happy (%)	Sad (%)	Surprise (%)	Neutral (%)
Angry	**91.1**	0.2	0.2	0	4.2	3.8	2.5
Disgust	5.3	**89.7**	3.0	2.6	1.0	0	2.4
Fear	0.24	2	**88.6**	3.4	5.9	1.16	0.7
Happy	1.5	0	2.1	**95.9**	0	0	0.5
Sad	1.0	5.3	4.2	0	**88.9**	0	0.6
Surprise	0.24	3.0	0.63	0	0.53	**94.6**	1.0
Neutral	2.12	3.8	3.27	0.3	0.79	0.44	**92.3**

Significant values are in bold.

### Loss and accuracy over time

It can be ensured that the loss reduces, and that the accuracy increases with each epoch. Training and test accuracy for training and validation losses collected using CNN for the FER2013 dataset are given in the Table 3. From the table, it can be ensured that as the epoch increases it shows a better accuracy rate for both the training and validation.

**Table 3:** Accuracy per epoch

Epoch	Training accuracy	Validation accuracy
1	34.14	44.04
2	47.87	50.40
3	53.05	53.91
4	56.14	56.76
5	58.87	58.32
6	60.35	56.98
7	62.28	59.32
8	63.88	61.44
	…	…
15	77.32	76.14
	…	…
30	96.48	91.05

### Accuracy and loss graph

**Figure 15:**
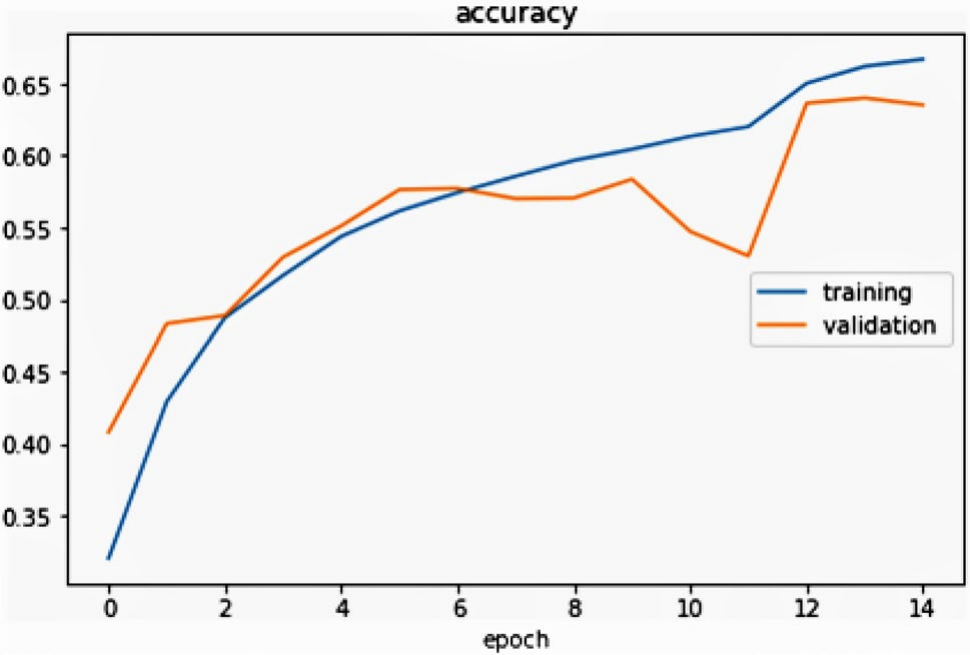
Graphical view of training and validation accuracy per epoch.

The authors recognize that the accuracy of training and validation are assessed to determine a model fitting. If there is a large difference between the two, the model is over-fitting. The accuracy of the validation should be equal to or marginally less than the accuracy of the preparation to be a better model. This work is also seen in Fig. 15, as the epoch improves the training accuracy is marginally higher than validation accuracy as the authors extended the layers and eventually introduced a few more convolution layers and several entirely related layers, rendering the network both larger and broader. It seems like the lack of preparation can still be smaller than the loss of validation.

**Figure 16:**
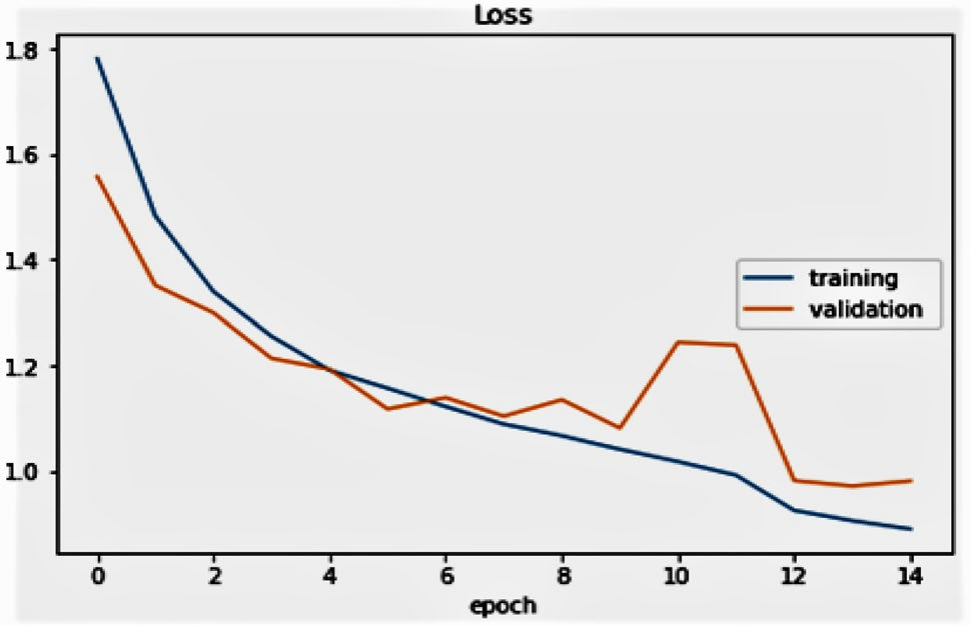
Graphical view of training and validation loss per epoch.

In Fig. 16, the authors show the corresponding training versus validation failure. This means that the training loss reduces as the epoch grows, and the validation loss increases. In addition, the validation data are always expected to decrease as the weights are adapted. Here, as the epoch grows in higher-order then we can expect a lower rate of validation loss than the training loss which we have already seen in the last stages of the figure. Therefore, this model is well suited to the training results.

### System validation

The generalization approach was used to further validate the system's performance. The word "generalization" refers to a model's capacity to respond to new data. This method can digest new data and generate accurate predictions after being trained. For generalization, this study employed the JAFFE and CK+ image datasets. The confusion metrics for the generalization findings are shown in Tables 4, and 5, respectively.

**Table 4:** Confusion matrix of 7 class facial emotion recognition results obtained by ConvNet with LBP on the JAFFE dataset.

	Angry (%)	Disgust (%)	Fear (%)	Happy (%)	Sad (%)	Surprise (%)	Neutral (%)
Angry	**92.10**	2.32	0	0	2.58	0	3.0
Disgust	5.42	**88.48**	3.42	0	2.68	0	0
Fear	0	1.13	**89.19**	0	5.48	4.25	0
Happy	0	0	0	**95.54**	2.12	0	2.34
Sad	1.45	2.29	1.68	1.21	**91.88**	0	0.49
Surprise	0	0	1.66	2.13	1.54	**94.67**	0
Neutral	1.12	3.8	1.27	0.3	0.79	0.44	**92.32**

Significant values are in bold.

**Table 5:** Confusion matrix of 7 class facial emotion recognition results obtained by ConvNet with LBP on the CK+ dataset.

	Angry (%)	Disgust (%)	Fear (%)	Happy (%)	Sad (%)	Surprise (%)	Neutral (%)
Angry	**98.61**	0	1.33	0	0.96	0	0
Disgust	0	**96.46**	0.27	0	1.97	2.30	0
Fear	0	0	**99.60**	0	0	0.40	0
Happy	0.31	1.95	0.32	**97.51**	0.30	0	1.61
Sad	1.11	1.29	0	1.10	**96.40**	0	1.03
Surprise	0.25	0	0	0.23	1.89	**97.28**	0.35
Neutral	1.12	1.80	1.27	1.97	2.17	0.42	**98.25**

Significant values are in bold.

Tables 4 and 5 shows that, with the exception of sadness, 7-class facial expressions are very accurately recognized, with an accuracy of above 92%. Here, we find that the efficiency of recognition is 92.05% for JAFFE dataset and 98.13% for CK+ dataset. A comparison of our results with those of related state of art methods are shown in Table 6**.**

**Table 6:** Comparison among existing methods in the Facial Emotion Recognition.

References	Methods	Dataset	Accuracy
Zhou et al.^49^	CNN + MVFE-LightNet	FER2013	68.4%
Ziyang Yu et al.^50^	CNN + music algorithm	FER2013	62.1%
P. Ferandez et al.^51^	FERAtt	CK+	82.11%
N. Christou and N. Kanojiya^23^	CNN	FER2013	91%
F. Wang et al.^29^	EFDMs + EEG + CNN	EEG data on SEED	90.59%
		DEAP	82.84%
F. Nonis et al.^31^	3d approaches	BU-3DFE	60% to 90%
Ben Niu et al.^52^	SVM + LBP + ORB	JAFFE	88.5%
		CK+	93.2%
		MMI	79.8%
Ji-Hae Kim et al.^53^	LBP + deep neural network	CK+	96.46%
		JAFFE	91.27%
Sawardekar and Naik^54^	LBP + CNN	CK+	90%
Fei Wang et al.^28^	EEE based EFDMs	Cross datasets	82.84%
Hongli Zhang et al.^9^	CNN + image edge computing	FER2013 + LFW	88.56%
Ke Shan et al.^55^	KNN + CNN	JAFFE	76.74%
		CK+	80.30%
Pham and Quang^56^	CNN + FPGA	FER2013	66%
Guohang Zeng et al.^57^	Deep learning + handcrafted feature	CK+	97.35%
Shan Li and Deng^58^	Deep learning	All facial dataset	45% to 95%
Zuheng Ming et al.^59^	FaceLiveNet	FER2013	68.60%
		CK +	98%
Hussain and Balushi^35^	Deep learning	KDEF	88%
Smitha Rao et al.^32^	CNN + LSTM	CREMA-D	78.52%
		RAVDEES	63.35%
Khalid Bhatti et al.^60^	Deep features + extreme learning	JAFFE	92.4%
		CK	91.4%
		FER2013	62.7%
Proposed method	Fusion features (CNN + LBP + ORB) + ConvNet	FER2013	91.01%
		JAFFE	92.05%
		CK +	98.13%

## Conclusion and future work

Fusion features were employed in the proposed study, and the input data were fused using LBP, ORB and CNN features. Based on the CK+ dataset, the proposed technique was found to have a better accuracy (98.13%) than other recent methods. In comparison to the current techniques, the other datasets equally obtained the best accuracy. The FER2013 also outperforms the previous studies in terms of classification accuracy. This method will be the same for trials using the MMI database as all of the facial expression pictures in the three datasets were taken from video sequences. As a result, the classification accuracy will be comparable. The identification rate will be better if we apply five or ten-fold cross validation techniques.

The authors have found seven distinct and unique emotion classes (fear, happy, angry, disgust, surprise, sad and neutral) in this article for emotion classification. There is no overlap between groups as our model perfectly fits the data. The presented research has achieved a very good average classification accuracy of emotions over 92% and also achieved 96% for random division of data in a minimal number of epochs. Based on the results from existing research studies, our model has been reflecting better in the real-time. The authors, however, plan to work with complex type or mixed group of emotions, such as shocked with pleasure, surprised by frustration, dissatisfied by anger, surprised by sorrow, and so on.

In this analysis, the authors conclude that due to certain variables, their proposed model is only on average. For the future, we will continue to focus on enhancing the consistency of each CNN model and layer and also try to examine new feature or method to be fused with CNN. The future study involves examining multiple forms of human variables such as personality characteristics, age, and gender that affect the efficiency of emotion detection. Because of the increasing availability of large medical data, machine learning techniques are being used to reveal hidden healthcare trends^61–67^. Deep neural networks, in particular, have recently been employed in healthcare applications^68^. Therefore, the proposed model has a great potential to be applicable on healthcare imaging data analysis.

Furthermore, the authors also focus on the mental or emotional stuff of a man or woman which helps as a consultant and leads the situation depending on behavioral things. Apart from this, we will attempt to refine the model more accurately such that a more natural method of recognition of facial expression can be provided.

## Data Availability

Extended Cohn–Kanade (CK+) and FER2013 datasets are publicly available datasets. Japanese Female Facial Expression (JAFFE) is not publicly available; however, we have been granted access to JAFFE dataset from publisher/author for training and testing purpose of our model.
